# Undocumented translocations spawn taxonomic inflation in Sri Lankan fire rasboras (Actinopterygii, Cyprinidae)

**DOI:** 10.7717/peerj.6084

**Published:** 2018-12-20

**Authors:** Hiranya Sudasinghe, Jayampathi Herath, Rohan Pethiyagoda, Madhava Meegaskumbura

**Affiliations:** 1 Department of Molecular Biology & Biotechnology, University of Peradeniya, Peradeniya, Sri Lanka; 2 Postgraduate Institute of Science, University of Peradeniya, Peradeniya, Sri Lanka; 3 Guangxi Key Laboratory of Forest Ecology & Conservation, College of Forestry, Guangxi University, Nanning, China; 4 Ichthyology Section, Australian Museum, Sydney, Australia

**Keywords:** mtDNA, *Rasboroides*, Allometry, Biodiversity hotspot, Integrative taxonomy, Systematics, Translocations, Species boundaries, Freshwater fish, Genetic distance

## Abstract

A recent (2013) taxonomic review of the freshwater-fish genus *Rasboroides*, which is endemic to Sri Lanka, showed it to comprise four species: *R. vaterifloris*, *R. nigromarginatus*, *R. pallidus* and *R. rohani.* Here, using an integrative-taxonomic analysis of morphometry, meristics and mitochondrial DNA sequences of *cytochrome b* (*cytb*) and *cytochrome oxidase subunit 1* (*coi*), we show that *R. nigromarginatus* is a synonym of *R. vaterifloris*, and that *R. rohani* is a synonym of *R. pallidus.* The creation and recognition of unnecessary taxa (‘taxonomic inflation’) was in this case a result of selective sampling confounded by a disregard of allometry. The population referred to *R. rohani* in the Walawe river basin represents an undocumented trans-basin translocation of *R. pallidus*, and a translocation into the Mahaweli river of *R. vaterifloris*, documented to have occurred *ca* 1980, in fact involves *R. pallidus.* A shared haplotype suggests the latter introduction was likely made from the Bentara river basin and not from the Kelani, as claimed. To stabilize the taxonomy of these fishes, the two valid species, *R. vaterifloris* and *R. pallidus*, are diagnosed and redescribed, and their distributions delineated. We draw attention to the wasteful diversion of conservation resources to populations resulting from undocumented translocations and to taxa resulting from taxonomic inflation. We argue against translocations except where mandated by a conservation emergency, and even then, only when supported by accurate documentation.

## Introduction

With a standard length that rarely exceeds 35 mm, *Rasboroides* and *Horadandia* are the only two genera of miniaturized cyprinid fishes to occur in Sri Lanka (Britz & Conway, 2009). *Horadandia* are distributed across the open lowland floodplains of Sri Lanka and south-western India, whereas *Rasboroides* are endemic to streams draining the rain forests of the island’s south-western quarter (Pethiyagoda, 1991). Known as Fire Rasboras in the ornamental-fish trade, the gentle disposition, attractive coloration and diminutive size of these fishes have made them a favourite among aquarists. The genus *Rasboroides* was considered to comprise of only a single species, *R. vaterifloris* (Deraniyagala, 1930), until Meinken (1957), based on aquarium specimens exported from Sri Lanka, described a second species, *R. nigromarginata.* Shortly thereafter, Deraniyagala (1958) treated *R. vaterifloris* as consisting—in effect—of five infraspecific taxa: *R. v. vaterifloris*, *R. v. nigromarginatus*, *R. v. pallidus*, *R. v. ruber*, and *R. v. rubioculis.*

**Figure 1:**
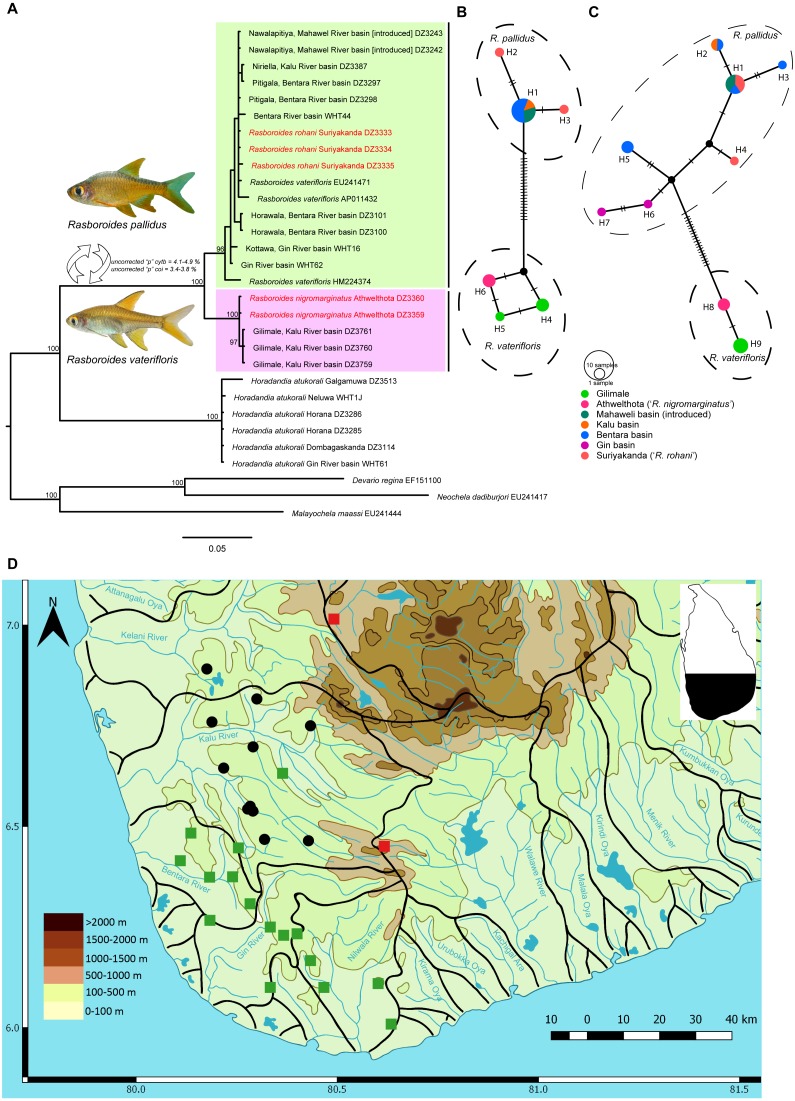
A Phylogram, haplotype networks for two genes and distribution map for species of *Rasboroides* in Sri Lanka. (A), Phylogram based on Bayesian inference for the *cytb* dataset for species of *Rasboroides* in Sri Lanka. Numbers above represent the Bayesian Posterior Probabilities. The scale bar represents number of changes per site; (B–C) TCS Haplotype network for species of *Rasboroides* in Sri Lanka based on the analysis of B, 669 bp fragment of the *coi*, and C, 609 bp fragment of the *cytb* genes. The sizes of the circles are proportional to the number of individuals sharing a given haplotype. The number of mutational steps are indicated by hatch marks. The black circles are hypothetical nodes; D, Distribution of *R. vaterifloris* (circles) and *R. pallidus* (squares) in Sri Lanka. The translocated populations of *R. pallidus* (red), and natural wild populations of *R. pallidus* (green).

Recently, Batuwita, De Silva & Edirisinghe (2013) in a taxonomic review of the genus *Rasboroides*, recognized three of these taxa (*R. vaterifloris*, *R. nigromarginatus* and *R. pallidus*) as valid at the rank of species, and relegated the remaining two to the synonymy of *R. pallidus*. While these three nominal species are distributed across the Kelani to the Nilwala river basins (Fig. 1D), Batuwita, De Silva & Edirisinghe (2013) described also a new species, *R. rohani*, from a localized population in the Walawe basin. This population too, was sampled by us in the course of fieldwork associated with a taxonomic assessment of the cypriniform fishes of Sri Lanka.

Several factors combine to make *R. rohani* unusual in comparison with the other species of *Rasboroides*. First, the genus *Rasboroides* had not previously been recorded from the Walawe basin (Deraniyagala, 1958; Senanayake, 1980; Pethiyagoda, 1991). Further, the only known population of *R. rohani* occurs at an elevation of ca 980 m asl, substantially higher than all other natural populations of *Rasboroides*, which occur in the approximate elevation range 30–230 m asl.

While these factors give rise to suspicion that *R. rohani* might in fact represent an undocumented introduction, Batuwita, De Silva & Edirisinghe (2013) nevertheless unambiguously distinguished this species from the three other species of *Rasboroides* they recognized, by an apparently robust suite of morphological characters. The recognition of a new species, however, results from testing the hypothesis that its phenotype and/or genotype, as represented by the examined sample of its population, differs sufficiently from those of congeneric species as to support its novelty according to one or more species concepts (Gaston & Mound, 1993; Winston, 1999). Here we test this hypothesis for *R. rohani* based on topotypic material collected by us, employing both morphological and molecular analyses, and show that *R. rohani* in fact represents an undocumented translocation of *R. pallidus.*

In their review, Batuwita, De Silva & Edirisinghe (2013) omitted to examine another population of *Rasboroides* in the vicinity of Ginigathena in the Mahaweli basin, from which the genus was absent until an introduction was made by F. R. Senanayake and P. B. Moyle in 1981 (Wikramanayake, 1990; *Moyle, in Pethiyagoda, 1991:36*). Although of uncertain provenance, this population is now well established and occurs across a range of some 5 km^2^. Here we show that this too, is the result of a translocation of *R. pallidus*. We further show also that *R. nigromarginatus*, considered to be a valid species by Batuwita, De Silva & Edirisinghe (2013), is in fact a synonym of *R. vaterifloris.* Finally, given the taxonomic problems manifestly resulting from the work of Batuwita, De Silva & Edirisinghe (2013), we diagnose and redescribe the two species of *Rasboroides* shown by our analyses to be valid: *R. vaterifloris* and *R. pallidus*.

## Materials & Methods

### Ethics statement

Permission to conduct field work was obtained from the Department of Wildlife Conservation (permit no. WL/3/2/59/14) and Forest Department (permit no. R&E/RES/NFSRCM/14-16-4) of Sri Lanka. Methods of specimen collection, euthanisation (using MS-222 Tricaine methanesulfonate), tissue sampling and fixation were approved by the ethical committee of the Postgraduate Institute of Science, University of Peradeniya, at its 27th meeting held on 4 August 2017.

### Metrics & meristics

Methods for measurements and counts follow Sudasinghe et al. (2018), except that the lateral-line scale count is given as the number of pored lateral-line scales + the scales between the last pored scale and the base of the hypural plate + the scales on the caudal-fin base. Scales in transverse line were counted diagonally to include the scales between the dorsal-fin origin and the lateral line row, +1, plus the scales between the lateral-line row and the origin of the anal fin. All measurements and counts were taken on the left side of specimens whenever possible. Head length and body measurements are represented as proportions of standard length; and subunits of the head as proportions of head length. Values in parentheses after a count represent the frequency of that count. The names *Rasboroides nigromarginatus* and *R. rohani* as used in this text refer to the populations to which these names were applied by Batuwita, De Silva & Edirisinghe (2013).

### Material

Specimens referred to in the text are deposited in the collections of the Natural History Museum, London (BMNH); the Zoological Museum Hamburg (ZMH); the Department of Molecular Biology and Biotechnology, University of Peradeniya (DZ); and the Wildlife Heritage Trust of Sri Lanka (WHT), now at the National Museum (NH) of Sri Lanka.

### Morphometric analysis

Sex was determined by the presence of tubercles on the anterior margin of the pectoral fin in males. Log-transformed measurements were checked for normality using a Shapiro–Wilk test. Following the results of the Shapiro–Wilk test, independent sample *t*-tests were carried out to test whether the morphological measurements were independent of sexual dimorphism in each putative species. In order to remove the effect of size-allometry, measurements were standardized by using the equation

\begin{eqnarray*}{M}_{s}={M}_{o}{ \left( \frac{{L}_{s}}{{L}_{o}} \right) }^{b} \end{eqnarray*}

where *M*_*s*_ is the standardized measurement, *M*_*o*_ is the measured character length, *L*_*s*_ is the overall (arithmetic) mean standard length for all individuals from all populations of all putative species, *L*_*o*_ is the standard length of each specimen, and *b* is estimated for each character from the observed data by using the allometric-growth equation *M* = *aL*^*b*^, where *b* is the gradient of regression of log*M*_*o*_ on log*L*_*o*_ (Elliott, Haskard & Koslow, 1995). The size-corrected data were used to conduct a Principal Component Analysis (PCA) of a covariance matrix. All statistical analyses were carried out in the software PAST (Hammer, Harper & Ryan, 2001).

### Molecular analysis

DNA was extracted from ethanol-preserved fin clips or muscle tissues using a DNeasy Blood & tissue kit (Qiagen, Hilden, Germany) in accordance with the manufacturer’s protocols. Details of the specimens used in the molecular analysis are given in Table 1. The partial mitochondrial *cytochrome b* (*cytb*) and *cytochrome oxidase subunit 1* (*coi*) were amplified. PCR was carried out in 25 µl reactions, using 2 µl of template DNA, 12.5 µl of mastermix MangoMix™ (Bioline, London, UK), 0.5 µl (for *cytb*), 0.3 µl (for *coi*) of each primer and 9.5 µl (for *cytb*), and 9.9 µl (for *coi*) of deionized water. The primer pair CB-J-10933 (5′ TATG TTCT ACCA TGAG GACA AATA TC 3′: Simon et al., 1994) and BSF4 (5′ CTTC TACT GGTT GTCC TCCG ATTCA 3′); FishF1 (5′TCAA CCAA CCAC AAAG ACAT TGGC AC3′) and FishR1 (5′TAGA CTTC TGGG TGGC CAA AGAA TCA3′: Ward et al., 2005) were used to amplify ∼615 bp and ∼670 bp of the *cytb*, and *coi* genes respectively. The thermal profile for *cytb* followed an initial denaturation at 95 °C for 5 min, followed by 35 cycles of denaturation at 95 °C for 40 s, annealing at 45 °C for 50 s, extension at 72 °C for 60 s and a final extension of 72 °C for 5 min; for *coi*, an initial denaturation at 95 °C for 2 min, followed by 35 cycles of denaturation at 94 °C for 0.5 min, annealing at 50 °C for 0.5 min, extension at 72 °C for 1 min and a final extension of 72 °C for 10 min. PCR products were visualized by an electrophoresis on 1.2% agarose gel and then purified using a PCR purification kit (Qiagen) and sequenced in both directions.

**Table 1:** Species included in the phylogenetic analysis with their localities, voucher references and GenBank accession numbers.

Species	Voucher	Location	Source	*cytb*	*coi*
*Rasboroides vaterifloris*	DZ3759	Sri Lanka: Kalu River basin, Gilimale	This study	MH780781	MH780767
*Rasboroides vaterifloris*	DZ3760	Sri Lanka: Kalu River basin, Gilimale	This study	MH780780	MH780766
*Rasboroides vaterifloris*	DZ3761	Sri Lanka: Kalu River basin, Gilimale	This study	MH780779	MH780765
*Rasboroides nigromarginatus*	DZ3359	Sri Lanka: Kalu River basin, Athwelthota	This study	MH780783	MH780769
*Rasboroides nigromarginatus*	DZ3360	Sri Lanka: Kalu River basin, Athwelthota	This study	MH780782	MH780768
*Rasboroides pallidus*	DZ3100	Sri Lanka: Bentara River basin, Horawala	This study	MH780790	MH780778
*Rasboroides pallidus*	DZ3101	Sri Lanka: Bentara River basin, Horawala	This study	MH780789	MH780777
*Rasboroides pallidus*	DZ3297	Sri Lanka: Bentara River basin, Pitigala	This study	MH780788	MH780776
*Rasboroides pallidus*	DZ3298	Sri Lanka: Bentara River basin, Pitigala	This study	MH780787	MH780775
*Rasboroides pallidus*	DZ3387	Sri Lanka: Kalu River basin, Niriella	This study	MH780786	MH780772
*Rasboroides pallidus*	DZ3242	Sri Lanka: Mahaweli River basin, Nawalapitiya	This study	MH780785	MH780771
*Rasboroides pallidus*	DZ3243	Sri Lanka: Mahaweli River basin, Nawalapitiya	This study	MH780784	MH780770
*Rasboroides pallidus*	WHT16	Sri Lanka: Gin River basin, Kottawa	This study	MH780794	N/A
*Rasboroides pallidus*	WHT44	Sri Lanka: Bentara River basin	This study	MH780795	N/A
*Rasboroides pallidus*	WHT62	Sri Lanka: Gin River basin	This study	MH780796	N/A
*Rasboroides pallidus*	CTOL00534	Not available	Genbank	HM224374	N/A
*Rasboroides pallidus*	NRM 50310	Not available	Genbank	EU241471	N/A
*Rasboroides pallidus*	CBM ZF 11546	Not available	Genbank	AP011432	AP011432
*Rasboroides rohani*	DZ3333	Sri Lanka: Walawe River basin, Suriyakanda	This study	MH780791	MH780774
*Rasboroides rohani*	DZ3334	Sri Lanka: Walawe River basin, Suriyakanda	This study	MH780792	N/A
*Rasboroides rohani*	DZ3335	Sri Lanka: Walawe River basin, Suriyakanda	This study	MH780793	MH780773
*Horadandia atukorali*	DZ3114	Sri Lanka: Kalu River basin, Dombagaskanda	This study	MH780801	MH780764
*Horadandia atukorali*	DZ3285	Sri Lanka: Kalu River basin, Remuna	This study	MH780800	MH780763
*Horadandia atukorali*	DZ3286	Sri Lanka: Kalu River basin, Remuna	This study	MH780799	MH780762
*Horadandia atukorali*	WHT1J	Sri Lanka: Gin River basin, Neluwa	This study	MH780798	MH780761
*Horadandia atukorali*	DZ3513	Sri Lanka: Mi Oya River basin, Galgamuwa	This study	MH780797	MH780760
*Horadandia atukorali*	WHT61	Sri Lanka: Gin River basin,	This study	MH780802	N/A
*Neochela dadiburjori*	NRM 50246	Not available	Genbank	EU241417	N/A
*Neochela dadiburjori*	LR1689	Not available	Genbank	NA	FJ753506
*Malayochela maassi*	NRM 50167	Not available	Genbank	EU241444	MF991139
*Devario regina*	LR1644	Not available	Genbank	EF151100	FJ753489

Sequenced data were checked and assembled in ChromasPro v1.34 (Technelysium Pty Ltd) and contig sequences of the two strands were prepared using MEGA v. 7.0 (Kumar, Stecher & Tamura, 2016). Additional available GenBank sequences were incorporated in to the phylogenetic analysis (Table 1). The *cytb* and *coi* contig dataset were constructed and aligned separately using ClustalW in MEGA v. 7.0 (Kumar, Stecher & Tamura, 2016), improved manually and translated and checked for premature stop codons or frameshift mutations. The uncorrected pairwise genetic distances for the putative species of *Rasboroides* for the two partial genes *cytb* and *coi* were calculated using MEGA. A barcoding gap analysis was conducted, separately for the *cytb* and *coi* gene sequences, using the Automatic Barcode Gap Discovery (ABGD) software of Puillandre et al. (2012) to delimit the putative species of *Rasboroides* by employing the K2P distance and a transition/ transversion ratio of 2.

Appropriate substitution model for the *cytb* and *coi* dataset was chosen by jModelTest 2 (Darriba et al., 2012) under the Bayesian Information Criterion (BIC). Bayesian inference for the two genes *cytb* (609 bp) and *coi* (669 bp) were carried out independently in MrBayes v3.2 (Ronquist et al., 2012). Four Metropolis-coupled Markov chain Monte Carlo (MCMCMC) chains were run for 1 million generations in two independent runs (chain temperature 0.1; sample frequency 100). Using Tracer (Rambaut et al., 2014), the first 100,000 generations were determined as burn-in and discarded. The frequency of the remaining clades in trees that were sampled every one hundred generations was used as an estimate of the posterior probabilities (PP) of those clades (Huelsenbeck et al., 2001). A maximum likelihood analysis was carried out for each gene using RAxML 8.0 (Stamatakis, 2014) implementing the GTRGAMMA model (see RAxML manual v8.2.X for justification) through the CIPRES Science Gateway (Miller, Pfeiffer & Schwartz, 2010). Clade support was assessed by rapid ML bootstrap analysis with 1,000 iterations. *Devario regina*, *Neochela dadiburjori* and *Malayochela maassi* were designated as outgroup taxa in all the analyses.

To estimate the divergence times between *Rasboroides* and *Horadandia*, and between sister-species pairs of *Rasboroides*, a Bayesian approach using BEAST v.10.0 (Suchard et al., 2018) was implemented. We used the average cyprinid *cytb* substitution rate of 0.0082 substitutions per site per million years and a standard deviation of 0.0025 substitutions per site per million years to calibrate the mitochondrial *cytb* tree under a normal prior (Rüber et al., 2004; Rüber et al., 2007). This substitution rate of *cytb* had been derived for European cyprinids in reference to two independent and well-dated geological events (Zardoya & Doadrio, 1999). Yule Process and strict clock model were specified as the tree prior and the clock type, respectively and two independent runs of 10 million generations, each sampling the Markov Chain Monte Carlo (MCMC) chain every 1,000 generations was carried out. Tracer was used to confirm convergence between the two runs and within each run and the first 0.1% the generations were discarded as burnin. The two runs were later combined and a maximum clade credibility (MCC) tree was constructed from the posterior sample of trees using TREEANNOTATOR, and visualized using FigTree v1.4.3 (http://tree.bio.ed.ac.uk/software/figtree).

Haplotype network reconstruction for the *cytb* and *coi* genes of putative species of *Rasboroides* was inferred by TCS network (Clement et al., 2002) in PopArt (Leigh & Bryant, 2015). TCS (Templeton, Crandall & Sing, 1992) has been used widely to infer genealogies of populations with low genetic divergences (Clement et al., 2002). GenBank sequences were not used since they derive from aquarium specimens of uncertain provenance. The nucleotide diversity (*π*) and neutrality tests Tajima’s D (Tajima, 1989) and Fu and Li’s F (Fu & Li, 1993) test statistics were computed using DNAsp v.6 (Rozas et al., 2017) for the species of *Rasboroides* in Sri Lanka.

## Results

### Phylogenetic analysis

The Bayesian and Maximum likelihood (Fig. S1) phylogenetic analyses for *cytb* and *coi* recovered similar topologies; hence, the Bayesian phylogram for *cytb*, which includes more samples, is given (Fig. 1A). The uncorrected pairwise distances obtained for putative species of *Rasboroides* for *cytb* and *coi* are given in Table 2. *Rasboroides rohani* and *R. nigromarginatus* are nested within the *R. pallidus* and *R. vaterifloris* clades, respectively. The minimum uncorrected pairwise *cytb* and *coi* distance between samples identified as *R. rohani* and *R. pallidus* is 0.0% for both, and between those identified as *R. nigromarginatus* and *R. vaterifloris*, is 0.2%. The GenBank sequences identified as deriving from *Rasboroides vaterifloris* (EU241471, AP011432, HM224374) are all apparently of *R. pallidus*. The population of *Rasboroides* introduced in the vicinity of Ginagathena (Wikramanayake, 1990) in the Mahaweli basin too, is *R. pallidus*. The intraspecific uncorrected pairwise distances for *cytb* and *coi* within *R. pallidus* (considering *R. rohani* as conspecific) are 0.0–2.4% (when HM224374 is included, 0.0–1.2% if HM224374 is excluded) and 0.0–0.3%, respectively, while those for *R. vaterifloris* (considering *R. nigromarginatus* as conspecific) are 0.0–0.2% and 0.0–0.3%, respectively. *Rasboroides pallidus* and *R. vaterifloris* differ genetically by a minimum uncorrected pairwise distance of 4.1% and 3.4% for *cytb* and *coi*, respectively. The uncorrected pairwise distances between *Rasboroides pallidus* and *H. atukorali* for the *cytb* and *coi* genes are 16.3% and 12.2%, respectively, while those between *R. vaterifloris* and *H. atukorali* are 17.9% and 13.0%, respectively.

**Table 2:** Intraspecific and interspecific percentage uncorrected pairwise genetic distances of the *coi* and *cytb* genes for putative species of *Rasboroides* in Sri Lanka.

%	*R. vaterifloris*		*R. nigromarginatus*		*R. pallidus*		*R. rohani*	
	*coi*	*cytb*	*coi*	*cytb*	*coi*	*cytb*	*coi*	*cytb*
*R. vaterifloris*	0.0–0.2	0.0						
*R. nigromarginatus*	0.2–0.3	0.2	0.0	0.0				
*R. pallidus*	3.4–3.5	4.3–4.9 (when HM224374 included), if not 4.3–4.7	3.4	4.1–4.7	0.0	0.0–2.4 (when HM224374 included), if not 0.0–1.2		
*R. rohani*	3.4–3.8	4.5–4.9	3.4–3.7	4.3–4.7	0.0–0.3	0.0–2.0 (when HM224374 included), if not 0.0–0.8	0.3	0.0–0.4

The Automatic Barcode Gap Detection (ABGD) algorithm, in which groups are empirically found to correspond with species (Puillandre et al., 2012), did not identify *R. nigromarginatus* and *R. rohani* as distinct groups. It did, however, identify <*R. pallidus* + *R. rohani* >and <*R. vaterifloris* + *R. nigromarginatus*>, as distinct groups, supporting their recognition as valid species.

### Divergence Timing

**Figure 2:**
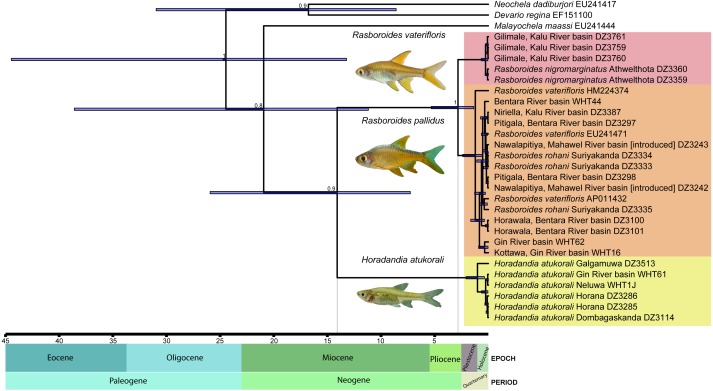
A time calibrated mitochondrial tree of *Horadandia arukorali* and species of *Rasboroides* in Sri Lanka. Bayesian posterior probabilities displayed for each major clade and node heights showing 95% highest posterior densities.

Based on the time-calibrated tree obtained (Fig. 2), the basal split between the common ancestor of *Horadandia* and *Rasboroides* occurred ∼14 mya, in the mid-Miocene, while that between *R. vaterifloris* and *R. pallidus* occurred ∼2.6 mya, in the late Pliocene (see Fig. 2 for 95% highest posterior densities).

### Reconstruction of the haplotype network

The TCS networks for the *coi* (Fig. 1B) and *cytb* (Fig. 1C) genes formed two clearly-separated haplotype groups for *R. pallidus* and *R. vaterifloris*, with minimum 21 and 19 mutational steps, respectively. There was no sharing of haplotypes between the two species. The haplotype network reconstructed for *cytb* includes samples from all the river basins in which *Rasboroides* is encountered, other than the Kelani and Nilwala. Within, the *R. pallidus* haplotype group for *cytb*, two shared (H1, H2) and five unique haplotypes (H3–H7) are identified. One sample from the population identified as *R. rohani* formed a unique haplotype (H4), while the other two samples formed a shared haplotype with samples from the Bentara and Mahaweli basins (H1); samples from the Kalu and Bentara basins formed the other shared haplotype (H2). The other two samples from the Bentara (H3, H5) and Gin basins (H6, H7) formed four unique haplotypes. It is possible that the specimens of *R. pallidus* introduced to the Mahaweli basin near Ginigathena and the Walawe basin at Suriyakanda (*R. rohani*) descended from a population in the Bentara basin. Within the *R. vaterifloris* haplotype group for *cytb*, samples from Gilimale (H9) and Athwelthota (H8, *R. nigromarginatus*) formed two unique haplotypes. Populations of *R. pallidus* (13 sequences) included 11 segregating sites and six parsimony-informative sites, while populations of *R. vaterifloris* (five sequences) each included only a single segregating site and parsimony-informative site. The nucleotide diversity was greater in *R. pallidus* than in *R. vaterifloris* (0.00584 vs. 0.00099) and Tajima’s D-test, negative for *R. pallidus* (−0.76736) and positive for *R. vaterifloris* (1.22474), was nevertheless insignificant (*p* > 0.05) in both species. Fu and Li’s *F*-test too, while negative for *R. pallidus* (−0.59785) and positive for *R. vaterifloris* (1.15728), was insignificant (*p* > 0.02) for both species.

**Table 3:** Results of the Independent sample *t*-tests of log transformed morphological measurements between the sexes of putative species of *Rasboroides* in Sri Lanka. Significance was accepted at *P* ≤ 0.05. Independent sample *t*-tests cannot be computed to the dataset of *R. rohani* alone, since only two females are represented in our sample. Values in bold are significantly different.

	*R. pallidus* (considering *R. rohani* as conspecific), males (*n* = 22, 22.2–33.6 mm SL) vs females (*n* = 10, 22.2–29.4 mm SL)			*R. pallidus* (excluding *R. rohani*), males (*n* = 12, 22.2–32.5 mm SL) vs females (*n* = 8, 22.2–29.4 mm SL)			Topotypic *R. vaterifloris*, males (*n* = 3, 21.2–24.2 mm SL) vs females (*n* = 6, 21.6–25.4 mm SL)			Topotypic *R. nigromarginatus*, males (*n* = 4, 24.7–28.9 mm SL) vs females (*n* = 5, 21.7–26.1 mm SL)			*R. vaterifloris* (considering *R. nigromarginatus* as conspecific), males (*n* = 7, 21.2–28.9 mm SL) vs females (*n* = 11, 21.6–26.1 mm SL)		
	*t*	*p*	Significant (*p* < 0.05)	*t*	*p*	Significant (*p* < 0.05)	*t*	*p*	Significant (*p* < 0.05)	*t*	*p*	Significant (*p* < 0.05)	*t*	*p*	Significant (*p* < 0.05)
Standard length	1.580	0.124	no	0.907	0.376	no	1.204	0.267	no	1.443	0.192	no	0.541	0.595	no
Predorsal length	1.476	0.150	no	0.863	0.399	no	0.779	0.461	no	1.771	0.119	no	0.939	0.361	no
Postdorsal length	1.791	0.083	no	1.169	0.257	no	0.520	0.618	no	2.200	0.063	no	1.290	0.215	no
Preanal length	1.251	0.220	no	0.667	0.512	no	0.929	0.383	no	0.773	0.464	no	0.306	0.763	no
Prepelvic length	1.365	0.182	no	0.778	0.446	no	0.870	0.412	no	1.151	0.287	no	0.618	0.544	no
Caudal peduncle length	1.722	0.095	no	1.181	0.252	no	0.721	0.494	no	1.462	0.186	no	0.849	0.408	no
Caudal peduncle depth	**2.152**	**0.039**	**yes**	1.559	0.136	no	0.791	0.454	no	1.721	0.128	no	0.816	0.425	no
Body depth	**2.375**	**0.024**	**yes**	1.756	0.095	no	0.588	0.574	no	2.211	0.062	no	1.434	0.170	no
Dorsal fin height	**3.001**	**0.005**	**yes**	**2.506**	**0.022**	**yes**	1.047	0.329	no	**3.109**	**0.017**	**yes**	**3.107**	**0.006**	**yes**
Dorsal fin base length	**2.683**	**0.011**	**yes**	2.073	0.052	no	1.247	0.252	no	0.517	0.620	no	1.263	0.224	no
Anal fin height	**2.747**	**0.010**	**yes**	**2.969**	**0.008**	**yes**	0.530	0.611	no	**3.996**	**0.005**	**yes**	**3.091**	**0.007**	**yes**
Anal fin base length	**2.194**	**0.036**	**yes**	1.513	0.147	no	0.106	0.918	no	**3.448**	**0.010**	**yes**	**2.124**	**0.049**	**yes**
Pelvic fin height	**2.545**	**0.016**	**yes**	**2.257**	**0.036**	**yes**	0.696	0.508	no	**4.131**	**0.004**	**yes**	**2.804**	**0.012**	**yes**
Pectoral fin height	**3.394**	**0.001**	**yes**	**3.446**	**0.002**	**yes**	0.411	0.693	no	**2.816**	**0.025**	**yes**	**2.399**	**0.028**	**yes**
Head length	1.410	0.168	no	0.942	0.358	no	1.608	0.151	no	1.047	0.329	no	0.217	0.830	no
Head depth	1.396	0.172	no	0.940	0.359	no	2.027	0.082	no	1.902	0.098	no	0.654	0.522	no
Snout length	0.945	0.351	no	1.224	0.236	no	0.732	0.487	no	2.236	0.060	no	1.022	0.321	no
Eye diameter	1.286	0.208	no	0.931	0.363	no	0.192	0.852	no	1.302	0.234	no	0.950	0.356	no
Inter orbital width	1.994	0.055	no	1.789	0.090	no	1.099	0.307	no	2.150	0.068	no	0.896	0.383	no
Inter narial width	1.528	0.136	no	1.836	0.082	no	0.234	0.820	no	1.306	0.232	no	0.822	0.422	no

Within, the *R. pallidus* haplotype group for *coi*, the samples identified as *R. rohani* formed two unique haplotypes (H2, H3) while those from the Bentara, Kalu and Mahaweli basins formed a single shared haplotype (H1). Within the *R. vaterifloris* haplotype group for *coi*, samples from Gilimale (H4, H5) and Athwelthota (H6, *R. nigromarginatus*) formed three unique haplotypes. Populations of *R. pallidus* (nine sequences) included nine segregating sites but no parsimony-informative sites, while populations of *R. vaterifloris* (five sequences) included only two segregating sites and two parsimony-informative sites. The nucleotide diversity was similar in both *R. pallidus* and *R. vaterifloris* (0.00100 vs. 0.00179) and Tajima’s *D*-test, negative for *R. pallidus* (−1.51297) and positive for *R. vaterifloris* (1.45884), was nevertheless not significant (*p* > 0.05) in both species. Fu and Li’s *F*-test was negative for *R. pallidus* (−1.82046) and positive for *R. vaterifloris* (1.43161), but insignificant (*p* > 0.02) for both species.

### Statistical analysis

The Shapiro–Wilk test showed all the measurements to be normally distributed (*p* > 0.05) among the sexes of *R. pallidus*, *R. vaterifloris* and the population referred to as *R. nigromarginatus* by Batuwita, De Silva & Edirisinghe (2013). The results of the independent sample *t*-tests for each measurement of the species are given in Table 3. Standard length, predorsal length, postdorsal length, preanal length, prepelvic length, caudal peduncle length, head length, head depth, snout length, eye diameter, interorbital width and internarial width were not significantly different (*p* > 0.05) between the sexes for any of the putative species. When *R. rohani* and *R. nigromarginatus* were considered as conspecific with *R. pallidus* and *R. vaterifloris*, respectively, dorsal-fin height, anal-fin height, anal-fin base length, pelvic-fin height and pectoral-fin height were significantly different (*p* < 0.05) between the sexes of each species.

**Figure 3:**
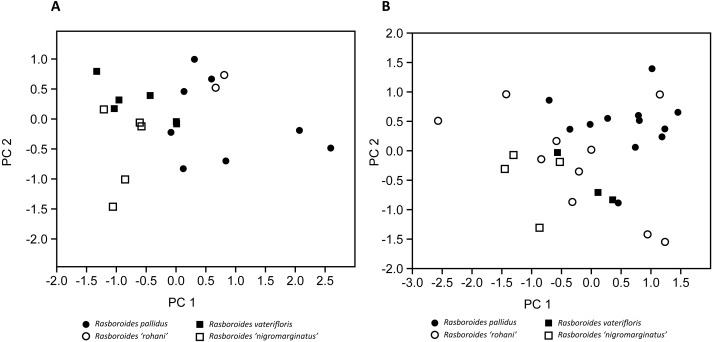
Plot of scores from the principal component analysis of size corrected measurements from putative species of (A) females, and (B) males of *Rasboroides* in Sri Lanka.

The results of the size corrected PCA are given in Fig. 3 and Table 4: in females, PC1 and PC2 explained 48.30% and 18.35% of the total variance, respectively. The females of *R. pallidus* and *R. rohani* are clustered together, while the females of *R. vaterifloris* and *R. nigromarginatus* form a separate cluster, with only a slight overlap between the two clusters. In males, PC1 and PC2 explained 33.82% and 19.35% of the total variance, respectively. The males of *R. pallidus* and *R. rohani* are clustered together, while the males of *R. vaterifloris* and *R. nigromarginatus* form a slightly overlapping cluster. When *R. rohani* and *R. nigromarginatus* are considered as conspecific with *R.  pallidus* and *R. vaterifloris*, respectively, the males form an overlapping cluster, while the females form almost non-overlapping cluster.

Hence, based on the multiple criteria assessed, we consider *R. rohani* and *R. nigromarginatus* to be junior synonyms of *R. pallidus* and *R. vaterifloris*, respectively.

**Table 4:** Component loadings in the principal component analysis of the size adjusted morphometric measurements of putative species of *Rasboroides* in Sri Lanka.

	Males		Females	
	PC 1	PC 2	PC 1	PC 2
Eigen value	0.988	0.565	1.063	0.404
Variance explained %	33.82	19.35	48.3	18.35
Predorsal length	0.1934	0.2198	0.3866	−0.1012
Postdorsal length	0.0567	0.2263	0.2073	−0.0098
Preanal length	0.2755	0.3472	0.3444	−0.4544
Prepelvic length	0.2928	0.2626	0.3176	−0.1383
Caudal peduncle length	−0.0061	−0.1260	−0.1504	0.0541
Caudal peduncle depth	0.0836	0.1191	0.1098	−0.0500
Body depth	0.4038	0.3988	0.4521	−0.0451
Dorsal fin height	0.4391	−0.3579	0.1367	0.3130
Dorsal fin base length	0.1058	−0.0217	0.0560	−0.2556
Anal fin height	0.5056	−0.5355	0.2670	0.6388
Anal fin base length	−0.0001	0.0247	0.1784	−0.0824
Pelvic fin height	0.2618	−0.154	0.1025	0.3328
Pectoral fin height	0.2171	0.0893	0.3201	0.2164
Head length	0.1237	0.0652	0.0555	−0.0255
Head depth	0.1717	0.2032	0.2948	0.0159
Snout length	0.0361	0.1567	0.1236	−0.0815
Eye diameter	0.0397	−0.0051	−0.0066	−0.0235
Inter orbital width	0.0563	0.0172	0.0462	−0.1117
Inter narial width	0.0350	0.0594	0.0579	−0.0043

### Species descriptions

#### General morphology

The following characters are common to *R. pallidus* and *R. vaterifloris*. Head and body laterally compressed. Body depth greatest at dorsal-fin origin. Dorsal profile of head concave behind level of eye; predorsal profile rising gently thereafter to origin of dorsal fin; postdorsal profile slightly concave. Ventral profile slightly convex up to origin of pelvic fin, straight from pelvic-fin origin to anal-fin origin, concave thereafter to base of caudal fin. Snout short, shorter than eye diameter, rounded in dorsal aspect, subtriangular in lateral aspect. Mouth terminal, rictus just passing vertical through anterior margin of eye. Symphysial knob present, minute, rounded, fitting into shallow groove on inner margin of upper jaw with mouth closed. Dorsal fin anterior margin straight, posterior margin slightly concave, its origin located just posterior to vertical through origin of pelvic fin. Tip of longest ray of dorsal fin, when adpressed, reaching beyond vertical through origin of anal fin. Pectoral fin originating ventrolaterally, immediately posterior to opercular membrane, its adpressed tip reaching beyond vertical through origin of pelvic fin. Adpressed tip of pelvic fin reaching vertical through base of 2nd branched anal-fin ray. Adpressed tip of anal fin reaching beyond midpoint of caudal peduncle.

#### Sexual dimorphism

**Figure 4:**
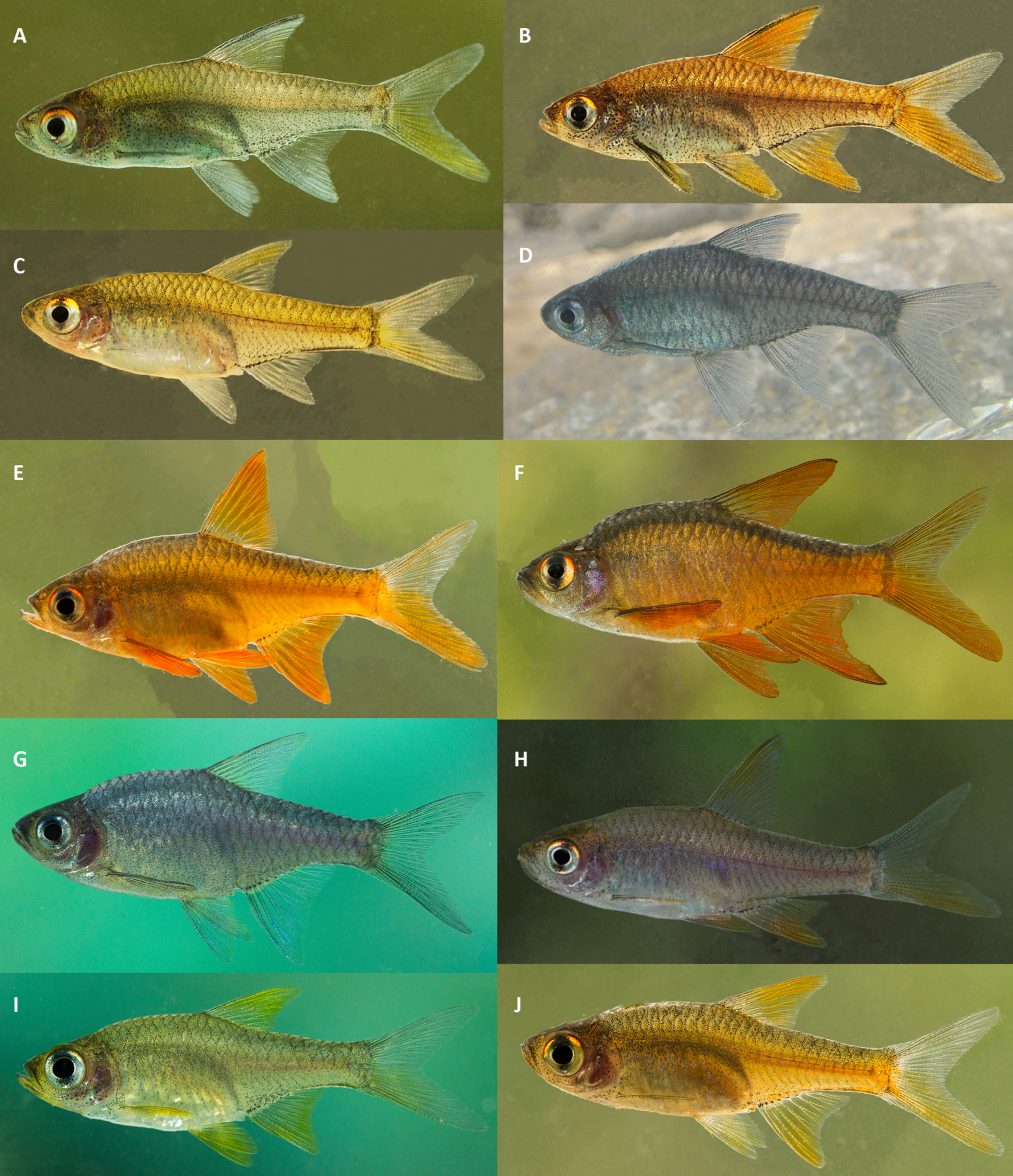
Live color pattern variation in A–D, *R. vaterifloris*; E–J, *R. pallidus*. (A) topotypes of *R. vaterifloris*, Kalu basin, Gilimale; (B–D) topotypes of population identified as *R. nigromarginatus* by Batuwita, De Silva & Edirisinghe (2013), Kalu basin, Athwelthota; (E) Bentara basin, Pitigala; (F) topotypes of population identified as *R. rohani* by Batuwita, De Silva & Edirisinghe (2013), Walawe basin, Suriyakanda; (G) Bentara basin, Yagirala; (H) Gin basin, Udugama; (I) Bentara basin, Yagirala; (J) Bentara basin, Pitigala. (A, B, D, E, F, G, H) males; (C, I, J) females. Specimens not collected.

Male specimens >21 mm SL have a series of conical tubercles along the anterior margin of the pectoral fin, a character absent in females. Male specimens >25 mm SL also have 7–10 bands of minute conical tubercles on the anterior lower jaw, reaching just beyond the rictus; and 1–3 bands of minute conical tubercles along the posterior margin of the preopercle, both characters absent in females. Some male specimens >25 mm SL possess a single row of tubercles beneath the eye, absent in females. Males generally with a greater body depth (31.8–44.4% SL vs. 31.7–35.5, *t* = 2.375, *p* = 0.024 in *R. pallidus*; 29–34.2% SL vs. 27.2–31.9, *t* = 1.434, *p* = 0.170 in *R. vaterifloris*). The male cranium shows a prominent concavity behind the level of the eye, and males have longer pectoral (23.7–30.5% SL vs. 21.5–25.3, *t* = 3.394, *p* = 0.001, in *R. pallidus*; 22.5–24.8% SL vs. 19.9–22.6, *t* = 2.399, *p* = 0.028 in *R. vaterifloris*), pelvic (21.4–31.6% SL vs. 21.1–25.4, *t* = 2.545, *p* = 0.016 in *R. pallidus*; 22.9–27.3% SL vs. 20-23.1, *t* = 2.804, *p* = 0.012 in *R. vaterifloris*), anal (24.5–40.8% SL vs. 22.9–31.5, *t* = 2.747, *p* = 0.010 in *R. pallidus*; 28.1–31.9% SL vs. 20.4–27.6, *t* = 3.091, *p* = 0.007 in *R. vaterifloris*) and dorsal fins (31.4–41.4% SL vs. 30.2–35, *t* = 3.001, *p* = 0.005 in *R. pallidus*; 32.6–36.8% SL vs. 29.1–33.1, *t* = 3.107, *p* = 0.006 in *R. vaterifloris*) than females of the respective species. Further, males of both *R. pallidus* and *R. vaterifloris* are more brightly colored than females (Fig. 4).

### *Rasboroides vaterifloris* Deraniyagala, 1930

**Table:** 

*Rasbora vaterifloris* Deraniyagala, 1930: 129
*Rasbora nigromarginata* Meinken, 1957: 65–68
*Rasbora vaterifloris* var. *nigromarginatus* Deraniyagala, 1958: 137
*Rasboroides nigromarginatus* (Meinken, 1957): Batuwita, De Silva & Edirisinghe, 2013

*Material examined* (all from Sri Lanka). DZ3768, topotypes of *R. vaterifloris*, 9, 21.2–25.4 mm SL, Kalu basin, Gilimale, 6°45′44.9″N 80°25′34.4″E; DZ3970, topotypes of ‘*R. nigromarginatus*’, 9, 21.7–28.9 mm SL, Kalu basin, Athwelthota, 6°32′35.7″N 80°16′38.0″E. Material not included in morphometric analysis: BMNH 1930.10.8.1, *Rasbora vaterifloris*, syntype, 25.7 mm SL, Kalu basin, near Illukvattai [Illukwatta] ferry on the Ratnapura to Gilimale road; WHT578, Kalu basin, Athwelthota.

*Diagnosis.* Males of *Rasboroides vaterifloris* can be distinguished from males of *R. pallidus* by having the unbranched rays of dorsal, anal, pectoral and pelvic fins black along their entire length, more distinctly evident in the last unbranched ray of the dorsal fin (vs. the mentioned rays being the same color as other rays; in preserved specimens, interradial membranes of dorsal, anal, pelvic and pectoral fins with distinct, scattered melanophores (vs. absent or vaguely present only around the beginning). Females of *R. vaterifloris* have a lesser body depth (27.2–31.9% SL vs. 31.7–35.5) than those of *R. pallidus*.

**Figure 5:**
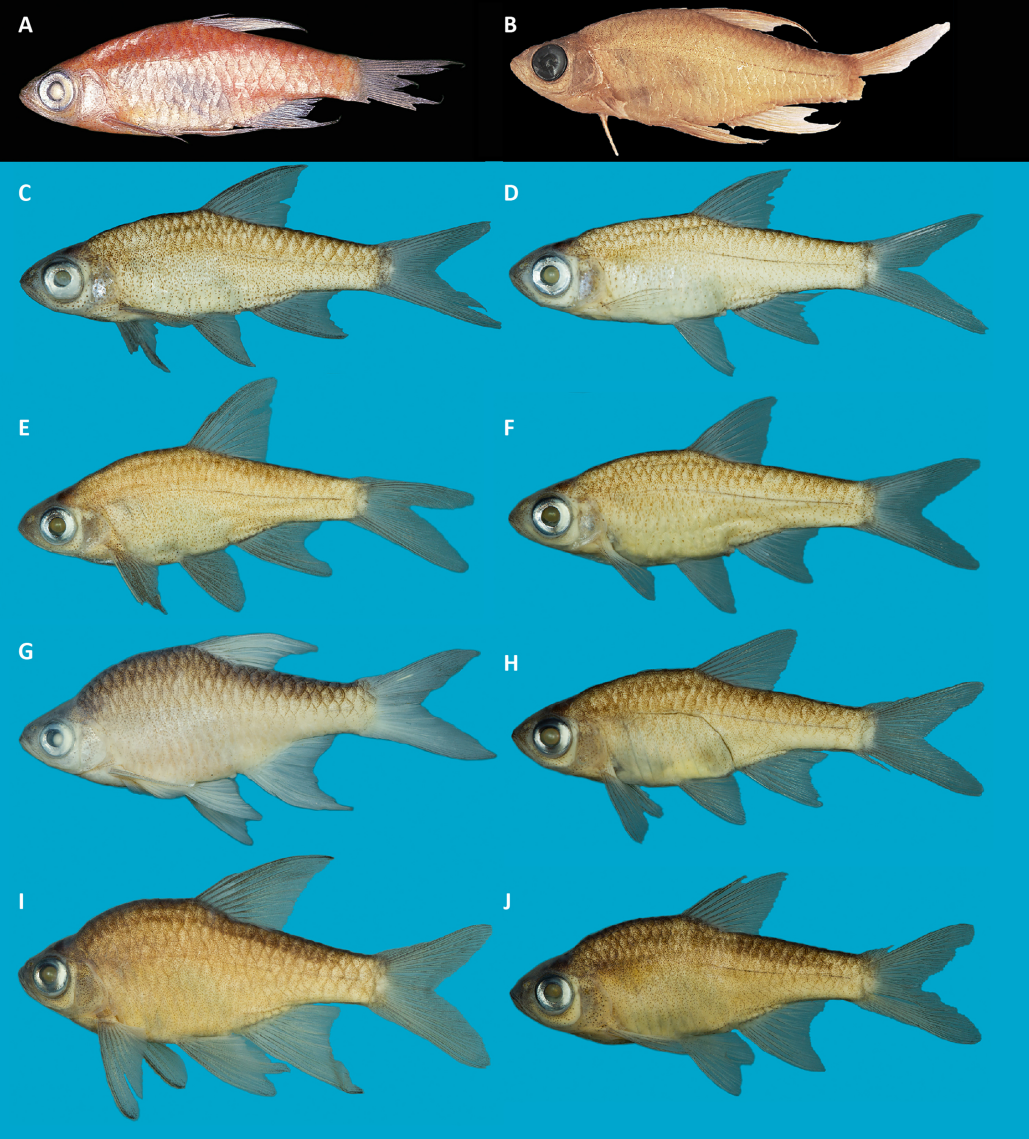
Depiction of *Rasboroides* species in preservation. (A) Syntype of *Rasboroides vaterifloris*, BMNH 1930.10.8.1, 25.7 mm SL, Kalu basin, Illukwatta; (B) holotype of *R. nigromarginatus*, ZMH 1207, 35.5 mm SL, ‘Ceylon’ (SriLanka); (C–D) topotypes of *R. vaterifloris*, Kalu basin, Gilimale: (C) male, DZ3768A, 24.2 mm SL, (D) female, DZ3768D, 24.4 mm SL; (E–F) topotypes of population identified as *R. nigromarginatus* by Batuwita, De Silva & Edirisinghe (2013), Kalu basin, Athwelthota: (E) male, DZ3970A, 28.9 mm SL, (F) female, DZ3970E, 26.1 mm SL; (G–H) *R. pallidus*: (G) male, DZ3972B, 31.0 mm SL, Bentara basin, Lewwanduwa, (H) female, DZ3973A, 29.4 mm SL, Bentara basin, Pitigala; (I–J) topotypes of population identified as *R. rohani* by Batuwita, De Silva & Edirisinghe (2013), Walawe basin, Suriyakanda: (I) male, DZ3971B, 31.9 mm SL, (J) female, DZ3971E, 28.9 mm SL.

**Table 5:** Morphometric data of *Rasboroides* vaterifloris (*n* = 18; DZ3768, DZ3970).

	*R. vaterifloris*				*R. nigromarginatus*				*R. vaterifloris* (considering *R. nigromarginatus* as conspecific)							
	males (*n* = 3)		females (*n* = 6)		males (*n* = 4)		females (*n* = 5)		males (*n* = 7)				females (*n* = 11)			
	min	max	min	max	min	max	min	max	min	max	mean	s.d	min	max	mean	s.d
Standard length	21.2	24.2	21.6	25.4	24.7	28.9	21.7	26.1	21.2	28.9			21.6	26.1		
In percent of standard length																
Predorsal length	51.9	53.8	50.9	53.4	52.7	54.0	50.2	53.5	51.9	54.0	53.0	0.8	50.2	53.5	52.0	1.0
Postdorsal length	51.9	53.4	50.0	52.1	52.3	53.9	50.0	52.2	51.9	53.9	53.0	0.7	50.0	52.2	51.3	0.8
Preanal length	60.4	62.4	58.4	63.2	58.7	61.6	59.5	65.1	58.7	62.4	60.9	1.2	58.4	65.1	61.4	2.0
Prepelvic length	45.8	47.2	44.7	46.7	44.2	46.6	45.1	46.7	44.2	47.2	45.9	1.0	44.7	46.7	45.8	0.7
Caudal peduncle length	23.6	25.5	23.2	24.7	23.6	24.9	22.4	26.8	23.6	25.5	24.4	0.7	22.4	26.8	24.1	1.2
Caudal peduncle depth	12.2	12.8	11.1	13.2	11.7	13.0	11.0	12.5	11.7	13.0	12.3	0.5	11.0	13.2	12.2	0.8
Body depth	29.0	31.0	27.2	31.3	31.5	34.2	27.2	31.9	29.0	34.2	31.6	1.7	27.2	31.9	29.7	1.6
Dorsal fin height	33.1	36.8	31.0	33.1	32.6	36.7	29.1	31.4	32.6	36.8	34.6	1.9	29.1	33.1	31.0	1.2
Dorsal fin base length	12.4	14.5	11.1	13.0	11.8	12.9	11.6	13.5	11.8	14.5	12.9	0.9	11.1	13.5	12.4	0.9
Anal fin height	28.1	28.6	24.1	27.6	28.6	31.9	20.4	27.2	28.1	31.9	29.6	1.5	20.4	27.6	25.0	2.1
Anal fin base length	15.0	15.8	13.4	15.9	15.3	16.6	13.5	15.2	15.0	16.6	15.7	0.6	13.4	15.9	14.5	0.8
Pelvic fin height	23.4	27.3	21.3	23.1	22.9	25.6	20.0	23.1	22.9	27.3	24.5	1.5	20.0	23.1	22.0	1.0
Pectoral fin height	23.2	24.8	20.9	22.6	22.5	24.6	19.9	22.5	22.5	24.8	23.8	0.8	19.9	22.6	21.5	1.0
Head length	26.1	28.8	26.0	28.1	25.2	28.4	25.4	27.2	25.2	28.8	26.5	1.5	25.4	28.1	26.9	0.8
Head depth	18.7	19.4	18.6	21.0	19.9	21.2	19.3	20.2	18.7	21.2	19.9	0.9	18.6	21.0	19.7	0.7
In percent of head length																
Snout length	18.1	25.4	19.5	23.4	21.5	28.6	20.0	23.6	18.1	28.6	22.8	3.6	19.5	23.6	21.3	1.4
Eye diameter	42.9	46.0	39.0	43.3	39.2	44.5	38.4	42.9	39.2	46.0	42.6	2.4	38.4	43.3	41.2	1.6
Inter orbital width	32.2	36.6	32.3	36.4	35.8	39.7	31.5	37.2	32.2	39.7	36.2	2.4	31.5	37.2	34.6	1.9
Inter narial width	19.7	22.3	17.0	21.3	19.2	24.4	19.2	21.9	19.2	24.4	20.9	1.9	17.0	21.9	19.9	1.4

*Description.* For general appearance, see Figs. 4A–4D, Figs. 5A–5F; morphometric data are provided in Table 5. Largest female 26.1 mm SL. Largest male 28.9 mm SL.

Dorsal fin with three unbranched and 7$ \frac{1}{2} $ (8) branched rays; first unbranched ray minute, less than $ \frac{1}{3} $ length of second; second unbranched ray stiff, less than half length of third. Anal fin with three unbranched and 6$ \frac{1}{2} $ (8) branched rays; first unbranched ray minute, less than $ \frac{1}{3} $ length of second; second unbranched ray stiff, less than half length of third. Pelvic fin with one simple and seven (8) branched rays. Origin of pelvic fin slightly anterior to vertical through origin of dorsal fin. Pectoral fin with one simple and 10 (1), 11 (6) or 12 (1) branched rays. Caudal fin forked, with 9+8 (7) branched rays in upper and lower lobe, respectively. Lower caudal-fin lobe slightly longer than upper.

Lateral body scales chaotically arranged. Lateral line incomplete, with 26 (2), 27 (3), 28 (1), 29 (1) or 30 (1) + 1 scales; pored scales 2 (2), 3 (4), 4 (2) or 5 (1). Scales in transverse series 8$ \frac{1}{2} $ (8). Circumpeduncular scales 10 (8). Predorsal scales 12 (4), 13 (1) or 15 (2). Prepelvic scales 15 (2), 16 (2) or 17 (3).

Coloration (in life) variable; at least three color morphs present. The male populations of *R. vaterifloris* from Gilimale, Parakaduwa, Madakada (Kalu basin) and Labugama (Kelani basin) are generally dull colored, with silvery-grey-brown body coloration, becoming lighter ventrally (Fig. 4A). Dorsal fin and lower lobe of caudal fin yellowish. Upper lobe of caudal fin, anal, pectoral, and pelvic fins dull colored. Male *R. vaterifloris* from Athwelthota, Runakanda, Pahiyangala, Kiriella (Kalu basin) laterally and dorsally bright golden-orange, becoming lighter ventrally (Fig. 4B). Dorsal, anal, pectoral, and pelvic fins golden orange. Lower lobe of caudal fin more golden orange than the upper lobe. A few individuals of *R. vaterifloris* from Athwelthota, Runakanda, Pahiyangala, Kiriella (Kalu basin) are greyish blue, becoming lighter ventrally (Fig. 4D). Females less brightly colored in all three morphs (Fig. 4C). Unbranched rays of dorsal, anal, pectoral and pelvic fins in males of all three color morphs black along their entire length, more distinctly in last unbranched dorsal-fin ray; these rays the same color as the remainder of the fin in females. Interradial membranes of dorsal, anal, pelvic and pectoral fins with scattered melanophores in males, absent in females. Upper half of sclera generally golden orange. Melanophores densely arranged on sclera above and below iris, giving appearance of a black bar on eye. Opercle tinged with red.

In preservative, overall body color brown, becoming lighter ventrally. Interradial membranes of dorsal, anal, pelvic and pectoral fins with scattered melanophores in males, uniform in females. Caudal fin hyaline. Last unbranched dorsal-fin ray of males blackish along its entire length.

### *Rasboroides pallidus* Deraniyagala, 1958

**Table:** 

*Rasbora vaterifloris pallida* Deraniyagala, 1958: 136.
*Rasbora vaterifloris ruber* Deraniyagala, 1958: 136.
*Rasbora vaterifloris rubioculis* Deraniyagala, 1958: 136.
*Rasboroides rohani* Batuwita, De Silva & Edirisinghe, 2013

*Material examined* (all from Sri Lanka). DZ3972, 5, 24.3–32.5 mm SL, Bentara basin, Walallawita, 6°24′53.3″N 80°06′35.0″E; DZ3973, 8, 22.8–27.4 mm SL, Bentara basin, Pitigala, 6°22′29.5″N 80°14′22.6″E; DZ3904, 7, 22.2–26.2 mm SL, Gin basin, Ma dola, 6°13′46.3″N 80°21′59.9″E; DZ3971, topotypes of ‘*R. rohani*’, 12, 25.9–33.6 mm SL, Walawe basin, Suriyakanda, 6°27′02.3″N 80°37′00.6″E. Material not included in morphometric data: 2013.22.01 NH, ‘*Rasboroides rohani*’, holotype?, 32.0 mm SL, Walawe basin, Suriyakanda; 2013.24.01 NH-2013.24.11 NH, ‘*R. rohani*’, paratypes, 11, 23.2–29.3 mm SL, Walawe basin, Suriyakanda; NH uncatalogued, ‘*R. rohani*’, holotype?, 32.2 mm SL, Walawe basin, Suriyakanda; NH uncatalogued, 2, 28.9–29.3 mm SL, ‘*R. rohani*’, paratypes?, Walawe basin, Suriyakanda; WHT30019, Mahaweli basin, Ginigathena; WHT30667, Gin basin, Udugama ela; WHT127, Bentara basin, Mahakalupahana, Horawala; WHT30049, Bentara basin, Bambarawana; WHT30607, Gin basin, Kottawa.

*Diagnosis.* The males of *Rasboroides pallidus* can be distinguished from the males of *R.  vaterifloris* by having the unbranched rays of dorsal, anal, pectoral and pelvic fins the same color as other branched rays (vs. black along their entire length); in preserved specimens, interradial membranes of dorsal, anal, pelvic and pectoral fins without distinct scattered melanophores throughout or with only minute, vague melanophores only around the beginning (vs. melanophores distinctly present). The females of *R. pallidus* have greater body depth (31.7–35.5% SL vs. 27.2–31.9) than females of *R. vaterifloris*.

*Description.* For general appearance, see Figs. 4E–4J, Figs. 5G–5J; morphometric data are provided in Table 6. Largest female 29.4 mm SL. Largest male 33.6 mm SL.

**Table 6:** Morphometric data of *Rasboroides pallidus* (*n* = 32; DZ3972, DZ3973, DZ3904, DZ3971).

	*R. pallidus*				*R. rohani*				*R. pallidus* (considering *R. rohani* as conspecific)							
	males (*n* = 12)		females (*n* = 8)		males (*n* = 10)		females (*n* = 2)		males (*n* = 22)				females (*n* = 10)			
	min	max	min	max	min	max	min	max	min	max	mean	s.d	min	max	mean	s.d
Standard length	22.2	32.5	22.2	29.4	25.6	33.6	28.8	28.9	22.2	33.6			22.2	29.4		
In percent of standard length																
Predorsal length	52.8	55.7	52.3	58.2	50.9	54.9	52.6	52.8	50.9	55.7	53.8	1.4	52.3	58.2	54.2	2.0
Postdorsal length	50.5	56.7	51.4	54.0	52.3	56.0	52.8	54.0	50.5	56.7	53.9	1.8	51.4	54.0	52.9	0.9
Preanal length	58.6	66.2	61.0	66.7	59.4	64.3	61.9	62.7	58.6	66.2	62.3	2.0	61.0	66.7	63.1	1.8
Prepelvic length	45.1	50.2	46.3	50.0	45.4	52.1	47.5	47.6	45.1	52.1	47.6	1.6	46.3	50.0	47.9	1.3
Caudal peduncle length	21.4	25.4	21.8	23.3	21.1	27.8	23.6	24.4	21.1	27.8	23.5	1.8	21.8	24.4	22.9	0.8
Caudal peduncle depth	11.8	14.8	11.1	14.0	11.8	13.9	11.8	13.2	11.8	14.8	13.1	0.7	11.1	14.0	12.6	1.0
Body depth	32.9	44.4	31.7	34.7	31.8	40.3	35.0	35.5	31.8	44.4	36.6	3.2	31.7	35.5	33.4	1.3
Dorsal fin height	32.2	37.9	30.2	35.0	31.4	41.4	32.3	33.6	31.4	41.4	36.1	2.4	30.2	35.0	32.7	1.6
Dorsal fin base length	12.0	14.2	10.6	14.2	11.5	15.0	11.9	12.9	11.5	15.0	13.4	0.9	10.6	14.2	12.4	1.2
Anal fin height	28.2	34.2	22.9	28.7	24.5	40.8	28.9	31.5	24.5	40.8	31.5	3.7	22.9	31.5	27.3	2.3
Anal fin base length	14.6	18.2	13.2	16.7	13.9	18.5	15.6	16.4	13.9	18.5	16.5	1.2	13.2	16.7	15.4	1.2
Pelvic fin height	22.8	27.4	21.1	24.3	21.4	31.6	23.2	25.4	21.4	31.6	25.7	2.3	21.1	25.4	23.1	1.3
Pectoral fin height	24.4	28.6	21.5	25.3	23.7	30.5	24.7	25.0	23.7	30.5	27.1	2.0	21.5	25.3	23.8	1.3
Head length	24.4	28.2	25.5	27.8	24.7	28.6	27.4	27.5	24.4	28.6	26.9	1.2	25.5	27.8	26.8	0.9
Head depth	20.6	24.0	20.7	23.6	19.6	23.7	21.5	22.3	19.6	24.0	22.0	1.2	20.7	23.6	21.9	1.0
In percent of head length																
Snout length	20.7	31.2	21.0	32.9	21.6	25.4	25.4	29.2	20.7	31.2	25.3	3.0	21.0	32.9	25.6	3.5
Eye diameter	36.1	44.5	38.5	43.1	36.6	44.5	38.0	39.3	36.1	44.5	39.9	2.7	38.0	43.1	40.7	1.6
Inter orbital width	34.3	40.0	29.2	39.5	33.0	36.8	33.0	36.8	33.0	40.0	36.0	1.8	29.2	39.5	35.1	3.0
Inter narial width	20.0	26.7	17.8	23.1	17.5	22.8	22.8	22.8	17.5	26.7	21.5	1.9	17.8	23.1	21.0	2.0

Dorsal fin with three unbranched and 7$ \frac{1}{2} $ (8) branched rays; first unbranched ray minute, less than $ \frac{1}{3} $ length of second; second unbranched ray stiff, less than half length of third. Anal fin with three unbranched and $6 \frac{1}{2} $ (11) branched rays; first unbranched ray minute, less than $ \frac{1}{3} $ length of second; second unbranched ray stiff, less than half length of third. Pelvic fin with one simple and seven (11) branched rays. Origin of pelvic fin slightly anterior to vertical through origin of dorsal fin. Pectoral fin with one simple and 10 (6) or 11 (5) branched rays. Caudal fin forked, with 9+8 (11) branched rays in upper and lower lobe, respectively. Lower caudal-fin lobe slightly longer than upper.

Lateral body scales chaotically arranged. Lateral line incomplete, with 24 (1), 25 (4), 26 (3), 27 (1), 28 (3), 29 (1) or 30 (1) + 1 scales; pored scales 2 (2), 3 (3), 4 (5), 5 (3) or 6 (1). Scales in transverse series 7$ \frac{1}{2} $ (3), 8 (5) or 8$ \frac{1}{2} $ (6). Circumpeduncular scales 8 (7) or 10 (7). Predorsal scales 10 (1), 11 (3), 12 (7), or 13 (2). Prepelvic scales 12 (1), 14 (2), 15 (6), 16 (1), 17 (1), 18 (1) or 19 (1).

Coloration (in life), variable, at least four color morphs present: orange, blue-grey, yellow and dull red (Figs. 4E–4J). Out of these, most common is the orange morph. However, in most of localities, 2–3 color morphs of *R. pallidus* generally occur together. Females less brightly colored in all morphs. Upper half of sclera generally golden orange. Melanophores densely arranged on sclera above and below iris, giving the appearance of a black bar on eye. Opercle tinged with red. Lower lobe of caudal fin more brightly colored than upper lobe.

In preservative, overall body color brown, becoming lighter ventrally. All fins hyaline.

### Habitat, distribution and natural history

Based on the present data, *R. vaterifloris* is restricted primarily to the Kalu basin, where it is recorded in several scattered localities in the elevation range of 31–169 m asl (Fig. 1D). However, we also recorded a small population of *R. vaterifloris* close to Labugama, which lies within the Kelani basin.

*Rasboroides pallidus* is more widely distributed than *R. vaterifloris*, being recorded from several localities within and between the Kalu, Bentara, Gin and Nilwala basins, in the elevation range 17–231 m asl. We have not encountered the two species in syntopy, though *R. pallidus* and *R. vaterifloris* occur in close proximity in some localities around Badureliya, near Mathugama, within the Kalu basin. The translocated population of *R. pallidus* in the Mahaweli basin occurs at an elevation of about 600 m asl and appears to have established itself successfully in the vicinity of Ginigathhena. The population described by Batuwita, De Silva & Edirisinghe (2013) as ‘*R. rohani*’ from Suriyakanda in the Walawe basin is evidently an undocumented translocation of *R. pallidus* (see ‘Discussion’). Both *R. vaterifloris* and *R. pallidus* usually occur in clear, shaded, slow-flowing streams and rivers. They are found often in large groups of more than 100 individuals, usually occupying the upper half of the water column, close to the margins.

## Discussion

### Translocations

As explained by P.B. Moyle in Pethiyagoda (1991:36) and Wikramanayake (1990), Senanayake and Moyle conducted an experimental study to establish refugial populations of several freshwater-fish species they considered to be threatened, by translocating them to a waterway near Ginigathena, in the upper regions of the Mahaweli basin. The fishes they targeted were all lowland-rainforest species—*Pethia reval* (as *Barbus cumingii*), *P. nigrofasciata* (as *B. nigrofasciatus*), *Puntius titteya* (as *B. titteya*) and *R. pallidus* (as *Rasbora vaterifloris*). Although the translocated fishes had been caught from the wild, their provenance was not in every case documented. However, the approximate locality and river basin of origin for three species is mentioned in Wikramanayake (1990), based on which the founder population of the translocated *Rasboroides* was stated to have been from Parakaduwa in the Kelani basin (Wikramanayake, 1990, table 2). Parakaduwa, however, drains to the Kalu basin, and Deraniyagala (1958) and Batuwita, De Silva & Edirisinghe (2013) both recorded *Rasboroides vaterifloris* from this locality. However, our molecular and morphological analysis shows the introduced Ginigathena population to be *R. pallidus*, casting doubt on the origin declared by Wikramanayake (1990). Based on the concordance of our haplotype network for *coi* and *cytb*, it seems plausible that the Ginigathena population of *R. pallidus* was founded by individuals drawn from the Bentara basin, in which it remains relatively common. In the almost four decades since these introductions were made, *R. pallidus*, *Pethia nigrofasciata*, *Pethia reval* and *Puntius titteya*, all of which were until then absent from the Mahaweli basin, have established substantial populations around Ginigathena. To this extent, the goal of establishing a refugial population of these species appears to have been met.

We show here that the Suriyakanda population of *Rasboroides* that Batuwita, De Silva & Edirisinghe (2013) named ‘*R. rohani*’ is in fact *R. pallidus.* As a result of selecting a series of *R. pallidus* and ‘*R. rohani*’ of different size ranges for either sex (see below), these authors evidently inferred morphological differences that should correctly have been attributed to allometry. What is more, the type material of ‘*R. rohani*’ declared by Batuwita, De Silva & Edirisinghe (2013) to be lodged in National Museum of Sri Lanka is discrepant with the specimens present in NH. The holotype specimen, according to Batuwita, De Silva & Edirisinghe (2013), measures 34.0 mm SL and was collected by S. Udugampala and R. Krishantha on 3 June 2013. The specimen catalogued as being the holotype of *R. rohani* (2013.22.01 NH), however, measures 32.0 mm SL and has been collected by S. Batuwita and S. Udugampola on 2 June 2012. There is a further uncatalogued specimen of ‘*R. rohani*’ labeled ‘holotype’, which measures 32.2 mm SL and has been collected by S. Udagampola and R. Krishantha on 1 July 2013. Similarly, the 11 paratypes of ‘*R. rohani*’ catalogued as 2013.24.01 NH-2013.24.11 NH measure 23.2–29.3 mm SL and have the same collector and date of collection as 2013.22.01 NH (the specimen presently registered as the holotype). According to Batuwita, De Silva & Edirisinghe (2013), WHT 9712 included 11 paratypes which measure 28.2–32.0 mm SL and were collected by R. Krishantha on 9 Feb 2012. The NH collection has in addition, two uncatalogued specimens of ‘*R. rohani*’ labeled ‘paratypes’, which measure 28.9–29.3 mm SL and were collected by S Udagampola and R Krishantha on 1 July 2013. Given the differences in the sizes, dates and collectors between those declared in Batuwita, De Silva & Edirisinghe (2013) and those actually registered at NH, it is impossible to identify with certainty the name-bearing type of *R. rohani*. Based on the topotypic material examined herein, and our molecular analysis, however, there is no doubt that the population Batuwita, De Silva & Edirisinghe (2013) described as ‘*R. rohani*’ derives from a translocated individuals of *R. pallidus*, the genus *Rasboroides* having been absent from the Walawe basin prior to this introduction.

In June 2018, a conservationist disclosed to two of us (HS and RP) on condition of anonymity that he had *ca* 2003 translocated, apparently in good faith, several specimens each of *Rasboroides* (evidently *R. pallidus*), *Devario pathirana*, *Puntius titteya*, *Pethia nigrofasciata* and *Malpulutta kretseri* to the stream at Suriyakanda that is the type locality of both ‘*R. rohani*’ and *Schistura madhavai* (Sudasinghe, 2017). Except for *Pethia nigrofasciata*, these species were previously not known from the Walawe basin (Deraniyagala, 1930; Senanayake, 1980; Pethiyagoda, 1991; present data). Our sampling of this stream suggests that *M. kretseri* and *D. pathirana* failed to establish there. *Puntius titteya* and *Pethia nigrofasciata*, however, still persist at this location, together with *R. pallidus*.

While translocation is recognized as a valid intervention in the conservation of freshwater fishes threatened in their natural habitat (Galloway et al., 2016), the translocation of freshwater organisms, especially if poorly planned or involving the transfer of species between basins, risks confronting the host community with threats including competition for resources, predation, and the unintentional transfer of pathogens, parasites and non-target species (Minckley, 1995; Arthington et al., 2004). The case of ‘*R. rohani*’ illustrates also the wasted time and effort, by Batuwita, De Silva & Edirisinghe (2013) and ourselves, first in describing a translocated species as new, and then in working to determine its provenance and, as it happens, ‘sink’ it. Had we not done so, ‘*R. rohani*’, by being a species with a highly restricted range, would almost certainly have been assessed as Critically Endangered and attracted substantial conservation attention, to the cost of more deserving threatened species. Indeed, Batuwita, De Silva & Edirisinghe (2013) advocated much the same, writing: “we hope that the present work will, by drawing attention to the previously unsuspected diversity within this genus and clarifying its taxonomy, lead to a fresh assessment of these fishes and actions to assure their conservation”.

### *Rasboroides pallidus* and ‘*R. rohani*’

Deraniyagala (1958) described three “races” of *Rasboroides* in addition to the forma typica, *R. v. vaterifloris*, namely, *R. v. pallidus*, *R. v. ruber* and *R. v. rubioculis*. Batuwita, De Silva & Edirisinghe (2013) considered these names to be simultaneous synonyms and, as first reviser, gave *R. pallidus* precedence over *R. rubioculis* and *R. ruber*. No type material is known for *R. pallidus*, *R. ruber* and *R. rubioculis* (Pethiyagoda, 1991: 336).

The population of *R. pallidus* at Suriyakanda, which Batuwita, De Silva & Edirisinghe (2013) described as ‘*R. rohani*’, is 980 m above sea level. This is substantially higher than the upper elevation limit of all naturally-occurring populations of *Rasboroides*, around 230 m asl. Deraniyagala (1958) suggested that *Rasboroides* may have had a wider distribution within the island during the Pleistocene. Had the species described as ‘*R. rohani’* been an isolated population persisting in a rainforest refugium, it would be expected to have a substantial genetic divergence from its sister species. For example, the *R. vaterifloris* from the Kalu basin differs genetically from the *R. pallidus* occurring in Kalu, Bentara and Gin basins by over 3% for both *coi* and *cytb* genes. However, ‘*R. rohani*’ and *R. pallidus* are genetically indistinguishable.

Consideration must be given also to the grounds on which Batuwita, De Silva & Edirisinghe (2013) misdirected themselves in considering the Suriyakanda population of *R. pallidus* to be a distinct species. Perhaps most significantly, the size range of the specimens of *R. pallidus* and ‘*R. rohani*’ examined by these authors contains no overlap: males, 21.5–24.6 mm SL in *R. pallidus,* vs. 25.3–35.5 mm SL in ‘*R. rohani*’; females, 20.2–20.7 mm SL in *R. pallidus,* vs. 23.0–30.8 mm SL in ‘*R. rohani’*. In our dataset, the females of *R. pallidus* are relatively smaller when compared with the females of ‘*R. rohani*’ (7 ex., 22.2–26.6 mm SL and 1 ex. 29.4 mm SL in *R. pallidus*, vs. 28.8–28.9 mm in ‘*R. rohani*’) and show differences in caudal-peduncle length (21.8–23.3% SL vs. 23.6–24.4, respectively), body depth (31.7–34.7% SL vs. 35.0–35.5, respectively) and anal-fin height (22.9–28.7% SL vs. 28.9–31.5, respectively). In the case of males of *R. pallidus* and ‘*R. rohani*’ examined by us, however, the series are of similar size (22.2–32.5 mm SL vs. 25.6–33.6, respectively) and relatively homogenous morphologically. We infer from this that the differences observed by Batuwita, De Silva & Edirisinghe (2013) between *R. pallidus* and ‘*R. rohani*’ were the result of allometry. Further, these authors distinguished ‘*R. rohani*’ from *R. pallidus* by the former having more scales in transverse line on body ($ \frac{1}{2} $8$ \frac{1}{2} $ vs. $ \frac{1}{2} $6–7$ \frac{1}{2} $) and more lateral-line scales (25–28 vs. 20–24). However, both these counts overlap in the material examined by us and the material available at NH.

Batuwita, De Silva & Edirisinghe (2013) noted also that ‘*R. rohani*’ attained a greater size (35.5 and 30.8 mm SL in males and females, respectively) than *R. pallidus* (24.6 and 20.7 mm SL, respectively). Indeed, the material examined by us too, reflects this condition, with a maximum size of 33.6 and 28.9 mm SL, respectively, for males and females of ‘*R. rohani*’, and 32.5 and 29.4 mm SL, respectively, for *R. pallidus*. We are unable to explain this phenomenon except by way of noting that the population of *R. pallidus* in the Yagirala Forest Reserve (Bentara basin) too, reaches ∼35mm SL. Unusually, at both these locations, *R. pallidus* is numerically the dominant cyprinid. We conjecture that its large size in such environments may be correlated with the paucity of larger and more numerous cofamilial competitors, possibly a case in which reduced interspecific competition enhances niche breadth (Robinson, Wilson & Margosian, 2000; Bolnick et al., 2010; Wootton, 2012).

### *Rasboroides vaterifloris* and ‘*R. nigromarginatus*’

The type locality of *R. vaterifloris* given by Deraniyagala (1930) is “near Illukvattai ferry on the Ratnapura to Gilimale road”. Consistent with this, Batuwita, De Silva & Edirisinghe (2013) identified the Gilimale population of *Rasboroides* as *R. vaterifloris*. *Rasboroides nigromarginatus* was described by Meinken (1957) based on aquarium specimens, and the exact origin of his material was unknown: “Heimat: Ceylon, genauer Fundort nicht zu ermitteln” (trans. “Home: Ceylon, exact location cannot be determined”). Batuwita, De Silva & Edirisinghe (2013), however, identified the population at Athwelthota in the Kalu basin as ‘*R. nigromarginatus*’ based primarily on coloration: “The coloration in life of the population we identify as *R. nigromarginatus* too, is exactly the same as that described by Meinken, including the characteristic blackened first ray of the dorsal, pectoral and pelvic fins, and the orange upper half of the sclera (Fig. 5A). We are confident therefore that our conception of *R. nigromarginatus* is the same as Meinken’s, with Atweltota being the likely type locality of this species”.

In his description of *R. vaterifloris*, however, Deraniyagala (1930) was careful to describe the color pattern as: “Dorsal fin with a black anterior edge, rest a bright orange as is the lower lobe of caudal...”. This description appears to have been overlooked by Batuwita, De Silva & Edirisinghe (2013), who described the male coloration of *R. vaterifloris* as “upper body golden brown, lightening on side to silvery, scattered with melanophores; belly silver. Dorsal, anal, pectoral and pelvic fins hyaline with scattered melanophores.” Further, they illustrated only a female (their fig. 3A). Deraniyagala (1958) further mentioned that the specimens of *R. vaterifloris* from Parakaduwa (from where Batuwita, De Silva & Edirisinghe (2013) examined material) as being “large with plenty of red on the upper half of the orbit [sic] upon the anal fin and lower lobe of the caudal”. At present, however, the *R. vaterifloris* at Gilimale are mostly dull-colored when compared with the population at Athwelthota. Similarly, dull-colored morphs of *R. vaterifloris* were also observed at Labugama (Kelani basin) and Madakada, Ingiriya (Kalu basin). Though the individuals of *R. vaterifloris* in these populations are less vividly colored, the last unbranched dorsal-fin ray of males shows the distinct black margin described in *R. vaterifloris* by Deraniyagala (1930) and in ‘*R. nigromarginatus*’ by Meinken (1957). This character is therefore of no value in distinguishing these nominal taxa.

It appears that Meinken (1957) was misled into describing *R. nigromarginatus* as a new species because the specimens he considered to be *R. vaterifloris* were in fact *R. pallidus*. This caused him to regard the specimens that were in fact *R. vaterifloris* as belonging to a new species. He stated: “Das zum Vergleich mitgeschickte junge Mannchen von *R. vaterifioris* ist im ganzen viel hiiher gebaut…der obere Teil des Auges ohne den orangeroten Glanz” (“the young male of the *R. vaterifloris*, which was sent for comparison, is much higher built…the upper part of the eye is without the orange-red gloss”). Meinken’s illustration of *R. vaterifloris* too, shows a fish with a higher profile, resembling *R. pallidus*.

In addition to neglecting to note Meinken’s confusion, Batuwita, De Silva & Edirisinghe (2013), as in the case of *R. pallidus* and ‘*R. rohani*’, were apparently misled by the different size-ranges of the series of *R. vaterifloris* and ‘*R. nigromarginatus*’ they examined: males, 23.9–28.4 mm SL in *R. vaterifloris*, vs. 26.2–30.2 mm SL in ‘*R. nigromarginatus*’; and females, 22.1–25.5 mm SL in *R. vaterifloris* vs. 26.5–27.3 mm SL in ‘*R. nigromarginatus*’. In our series too, the males of *R. vaterifloris* from the type locality (Gilimale) are smaller than those of ‘*R. nigromarginatus*’ sensu Batuwita, De Silva & Edirisinghe (2013) from Athweltota (21.2–24.2 mm SL, vs. 24.7–28.9). The proportional body depths in these series do not overlap: 29.0–31.0% SL in topotypical *R. vaterifloris* vs. 31.5–34.2 in ‘*R. nigromarginatus*’), as do not also the anal-fin height (28.1–28.6% SL in *R. vaterifloris* vs. 28.6–31.9 in ‘*R. nigromarginatus*’) and head depth (18.7–19.4% SL in *R. vaterifloris* vs. 19.9–21.2 in ‘*R. nigromarginatus*’). Females of *R. vaterifloris* and ‘*R. nigromarginatus*’ in our series were, however, similar in size (21.6–25.4 mm SL vs. 21.7–26.1, respectively) and did not differ in any of the mentioned proportions. It appears likely, therefore, that the proportional differences between *R. vaterifloris* and ‘*R. nigromarginatus*’ observed by Batuwita, De Silva & Edirisinghe (2013) were a coupling of selective sampling and allometric growth, not indicative of a morphometric signal.

Further, the minimum uncorrected pairwise distances for the partial genes *cytb* and *coi* between topotypical *R. vaterifloris* and ‘*R. nigromarginatus*’ are only 0.2%, in addition to which there appears to be no geographic barrier separating the two populations. We are confident, therefore, in referring ‘*R. nigromarginatus*’ to the synonymy of *R. vaterifloris*. The GenBank sequences earlier identified as *R. vaterifloris* (HM224374, EU241471, AP011432) were all shown to be *R. pallidus* when our newly generated sequences were included in the molecular analysis. Such species misidentifications are common in GenBank (Conte-Grand et al., 2017) and care should be taken when interpreting GenBank records with no specific specimen data.

## Conclusion

As a freshwater-fish genus endemic to Sri Lanka and restricted largely to streams draining the island’s dwindling rainforest estate, *Rasboroides* attracts considerable conservation attention. The National Red List (MOE, 2012) treats ‘*R. nigromarginatus*’ as Critically Endangered and *R. vaterifloris* as Endangered. The synonymy of these two nominal species demonstrated here allows their ranges to be combined, widening their extent of occurrence and area of occupancy and hence potentially lowering the threat-status of *R. vaterifloris.* Although ‘*R. rohani*’ has not as yet been assessed for conservation purposes, its restriction to a small population at a single locality would almost certainly have caused it to be ranked as Critically Endangered. Given that we show here that it represents only an undocumented translocation of *R. pallidus*, its population is now only of marginal conservation concern. Indeed, of the two valid species of *Rasboroides*, *R. pallidus* enjoys the wider range and hence warrants less conservation concern, especially given its successful translocation to two river basins (Mahaweli and Walawe) in which it did not previously occur.

In describing ‘*R. rohani*’ as a new species, Batuwita, De Silva & Edirisinghe (2013) were misled by apparently collecting only the largest specimens for their sample while neglecting to account for allometric growth. It is additionally regrettable that the type series of ‘*R. rohani*’ designated by these authors cannot be identified in the collection of the National Museum of Sri Lanka, in which it was stated to be deposited.

Both translocations referred to in this paper were made by well-meaning citizens but without the safeguards that should apply in such cases. Perhaps most egregiously, no records were published of the rationale for translocation or the precise identity and origin of the source population. We urge that any future attempts to introduce species to novel habitats be guided by IUCN/SSC (2013) and that the intentional release or introduction of species without legal sanction be prohibited in Sri Lanka.

##  Supplemental Information

10.7717/peerj.6084/supp-1 File S1 Raw data of *Rasboroides* Raw data of *Rasboroides* used in the morphological analyses along with sampling localities and voucher numbers.

10.7717/peerj.6084/supp-2 File S2 *coi* alignment *coi* alignment of *Rasboroides* used in the phylogenetic analysis

10.7717/peerj.6084/supp-3 File S3 Maximum Likelihood phyolograms Maximum Likelihood phylograms based on *cytb* and *coi* datasets for species of *Rasboroides* in Sri Lanka

10.7717/peerj.6084/supp-4 File S4 *cytb* alignment of *Rasboroides* *cytb* alignment of *Rasboroides* used in the phylogenetic analysis

## Additional Information and Declarations

### Field Study Permissions

The following information was supplied relating to field study approvals (i.e., approving body and any reference numbers): Field research permission was granted by the Department of Wildlife Conservation (DWC) Sri Lanka and the Forest Department of Sri Lanka (FD) (WL/3/2/59/14 & R&E/RES/NFSRCM/2016-04).

### DNA Deposition

The following information was supplied regarding the deposition of DNA sequences: The sequences generated for this study are available from the Genbank under accession numbers MH780760–MH780802.

### Data Availability

The following information was supplied regarding data availability: The morphological data set used in this study and the working DNA data set have been uploaded as Supplemental Files.
