# Supplementary material for: Undocumented translocations spawn taxonomic inflation in Sri Lankan fire rasboras (Actinopterygii, Cyprinidae)
Source: PeerJ. 2018 Dec 20;6:e6084. doi: 10.7717/peerj.6084 (PMC6304270; doi:10.7717/peerj.6084)
Supplement: File S3 — Maximum Likelihood phylograms based on cytb and coi datasets for species of Rasboroides in Sri Lanka [file peerj-06-6084-s003.pdf]

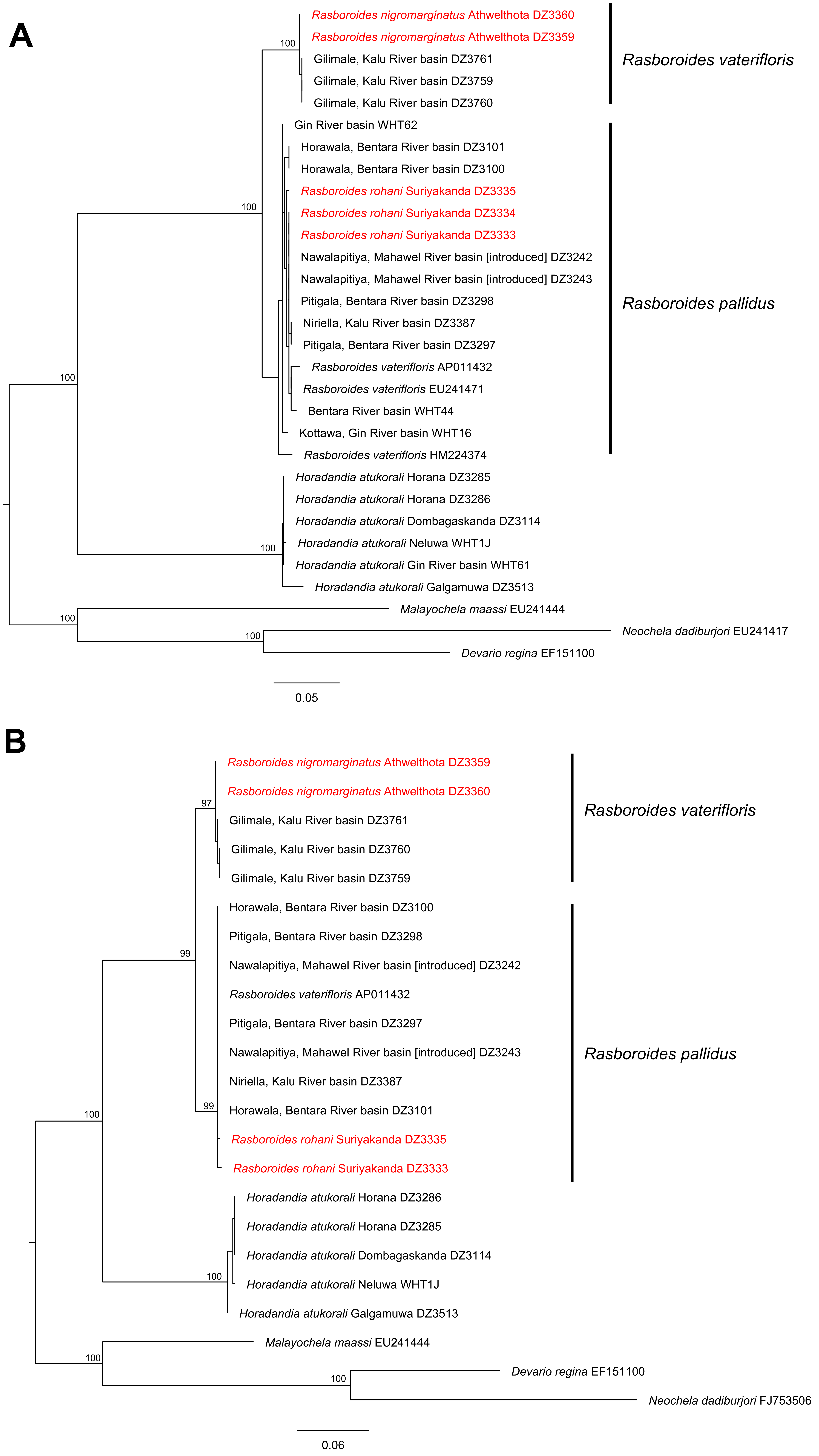

Supplement Figure 1. Maximum Likelihood phylograms based on **A**, *cytb* and **B**, *coi* datasets for species of *Rasboroides* in Sri Lanka. Numbers above nodes represent the ML bootstrap values. The scale bar represents number of changes per site.
